# Religious Coping, Depression and Anxiety among Healthcare Workers during the COVID-19 Pandemic: A Malaysian Perspective

**DOI:** 10.3390/healthcare9010079

**Published:** 2021-01-15

**Authors:** Soon Ken Chow, Benedict Francis, Yit Han Ng, Najmi Naim, Hooi Chin Beh, Mohammad Aizuddin Azizah Ariffin, Mohd Hafyzuddin Md Yusuf, Jia Wen Lee, Ahmad Hatim Sulaiman

**Affiliations:** 1 Department of Psychological Medicine, University Malaya Medical Centre, Kuala Lumpur 59100, Malaysia; sunjian872@gmail.com (S.K.C.); ben.franciscan@gmail.com (B.F.); 2 Department of Social Preventive Medicine, Faculty of Medicine, University of Malaya, Kuala Lumpur 50603, Malaysia; hanzhai2574@gmail.com; 3 Psychological Medicine Research Centre, University Malaya Medical Centre, Kuala Lumpur 59100, Malaysia; najmi.naim@ummc.edu.my; 4 Department of Primary Care Medicine, University Malaya Medical Centre, Kuala Lumpur 59100, Malaysia; hcbeh@ummc.edu.my; 5 Department of Emergency Medicine, University Malaya Medical Centre, Kuala Lumpur 59100, Malaysia; aizuddin@ummc.edu.my (M.A.A.A.); hafyzuddin@ummc.edu.my (M.H.M.Y.); 6 Faculty of Medicine, University Malaya, Kuala Lumpur 50603, Malaysia; 7 Department of Anesthesiology, University Malaya Medical Centre, Kuala Lumpur 59100, Malaysia; carmenlee0109@gmail.com

**Keywords:** anxiety, depression, COVID-19, healthcare workers, pandemic, religious coping

## Abstract

Anxiety and depression have been prevalent among Healthcare Workers (HCWs) amidst the COVID-19 pandemic. This study aims to evaluate the prevalence of anxiety and depression among HCWs amid the pandemic and their association with religious coping. A cross-sectional study design was applied. The scales utilized were Malay versions of the Brief Religious Coping Scale (Brief RCOPE M) and Hospital Anxiety and Depression Scale (HADS M). In total, 200 HCWs were recruited. HCWs scored higher in positive religious coping (mean: 21.33) than negative religious coping (mean: 10.52). The prevalence of anxiety and depression was 36.5% and 29.5%. Both positive and negative religious coping were significantly associated with anxiety (*p* < 0.01) and depression (*p* < 0.05, *p* < 0.01). Positive coping predicted reduction in anxiety (adjusted b = −0.15, *p* = 0.001) and log-transformed depression score (adjusted b = −0.019, *p* = 0.025). Negative coping predicted increment of anxiety (adjusted b = 0.289, *p* < 0.001) and log-transformed depression score (adjusted b = 0.052, *p* < 0.001). Positive religious coping is vital in reducing anxiety and depression among HCWs amid the pandemic. Strategies which increase positive religious coping and reduce negative religious coping must be emphasized to boost mental health among HCWs.

## 1. Introduction

The COVID-19 pandemic has wreaked chaos worldwide, affecting scores of people. As of 15th January 2021, a total of 147,855 positive cases of COVID-19 [1] were documented in Malaysia, including 578 deaths. Facing this critical challenge, healthcare workers (HCWs) from many nations, including Malaysia, relentlessly fought the pandemic. HCWs involved in the direct management of the patients infected with COVID-19 are susceptible to experiencing psychological symptoms. Regional studies in Asia documented the prevalence of anxiety and depressive symptoms among HCWs amid the pandemic to be as high as 8.7–44.6% and 5.3–50.6% [2,3,4,5,6], respectively.

Recent meta-analyses [4,5] assessing the risks of mental illnesses among HCWs amidst the pandemic found gender and occupational differences to be factors. Interestingly, females and nursing staff revealed higher levels of anxiety and depressive symptoms. Various risk factors have been identified as being associated with adverse mental health outcomes amongst the HCWs during the pandemic, such as social isolation, lack of support, battling on the frontline, fear of infecting colleagues or family members [7]. Furthermore, HCWs were especially prone to develop post-traumatic stress disorder (PTSD) or post-traumatic symptoms according to a recent review which focused on three Coronavirus outbreaks, including SARS 2003, MERS 2012 and COVID-19 [8]. In the same review, positive coping skills were found to be protective against developing PTSD. Positive coping strategies such as the application of humor, altruistic acceptance of the risks related to work [9] and confiding relationships were reported to be associated with lower rates of PTSD.

A recent review pointed out the importance of spirituality as a coping skill and in maintenance of psychological well-being for both patients and HCWs during the pandemic [10]. In the same review, spirituality was found to aid HCWs in coping with stress, encourage recovery, resilience and reduction in burnout. Another study in Brazil also revealed the significant role of religiosity and spirituality in reducing fear, sorrow and anxiety in relation to the COVID-19 pandemic and consequent social isolation [11].

Our study focuses on examining religious coping against adverse mental health outcome. Religion and mental health have a complex relationship. Pargament [12] described religious coping as a form of coping skill which utilizes religion in dealing with life adversities. Religious coping consists of positive and negative religious coping skills. Positive religious coping involves benefitting a favorable bond with God by praying or connecting to God during crises. On the other hand, negative religious coping refers to blaming God for one’s own hardship. Positive and negative religious coping have been associated with higher and lower levels of psychological health, respectively [13].

Malaysia is a country with a multireligious background, with Islam as the national religion. Other religions such as Christianity, Buddhism and Hinduism are practiced without restrictions. Hence, in a multireligious setting such as in Malaysia, it is crucial to assess the association of religious coping with mental health. The religion of Islam, specifically, has been documented as being protective against adverse mental health issues in nonpandemic settings [14]. In the face of SARS 2003 outbreak, religious beliefs [15] were found to be potentially protective towards developing PTSD amongst the HCWs. Hence, the current study is necessary to evaluate the possible protective effect of religious beliefs, specifically religious coping strategies, amid the COVID-19 pandemic.

Prior local studies in Malaysia, on the topic of religious coping and mental health, put their focus on psychiatric patients [16] and medical students [17], revealing the association between negative religious coping and poorer mental health outcomes in a nonpandemic setting. Internationally, similar studies which were conducted in different countries [18,19] consistently demonstrated significant associations between negative religious coping and worsened psychological well-being in a nonpandemic setting. Although there were recent studies amid the COVID-19 pandemic, which revealed the association of positive religious coping with a reduction in psychological morbidities [20,21], existing evidence showed a stronger association between negative religious coping and mental health outcomes.

This study centered on examining the association of both positive and negative religious coping with mental health amongst vulnerable HCWs during the pandemic. Knowledge on religious coping and its association with mental health could aid mental health professionals in providing more tailored risk stratification and intervention for the HCWs, particularly when encountering an unprecedented global crisis such as the COVID-19 pandemic. The prime objective of the study is to evaluate the association between religious coping, depression and anxiety amongst HCWs, within the Malaysian setting during the COVID-19 pandemic. The secondary objective is to determine the prevalence of depression and anxiety amongst the HCWs involved in managing the pandemic.

## 2. Materials and Methods

A cross-sectional study was commenced amongst HCWs in University Malaya Medical Centre (UMMC). UMMC is a premier university hospital in Malaysia, designated to treat COVID-19 patients, and situated in the heart of Kuala Lumpur, the epicenter of the COVID-19 pandemic in Malaysia. This study utilized a nonprobability convenient sampling method, and questionnaires were distributed to HCWs who consented for the study. The individuals who consented to be enrolled in the research were assessed based on the subsequent inclusion and exclusion criteria. In total, 200 HCWs who fulfill the inclusion criteria and fell short of the exclusion criteria joined the research.

### 2.1. Inclusion Criteria

- Malaysian healthcare workers presently working in UMMC.
- Healthcare workers must be managing patients suspected to have or infected with COVID-19.
- Aged 20 and above.
- Healthcare workers must have a religious belief.

### 2.2. Exclusion Criteria

- Refusal to join research.

Socio-demographic data were gathered using preconstructed questionnaires designated for the research. Religious coping was assessed via Malay translation of the Brief Religious Coping Scale (BRCOPE M). Depression and anxiety were evaluated via the Malay translation of Hospital Anxiety and Depressive Scale (HADS M). Both tools were validated to be used in Malaysian setting. This study was approved by the Medical Ethical Committee of UMMC (MREC 202044-8445).

### 2.3. Measurement Tools

#### 2.3.1. BRCOPE (M)

The BRCOPE was used to measure religious coping. It has seven positive coping measures and seven negative coping measures, respectively. Each item was coded from 1 to 4 in a four-point Likert scale. The scores for positive and negative coping therefore varied from 7 to 28. The scale was devised by Pargament [12] to evaluate the role of religion in dealing with life difficulties and crisis. The scale includes both positive and negative religious coping skills. The translated Malay version of this scale has Cronbach alphas of 0.87 and 0.88, respectively, for positive religious coping and negative religious coping [22].

#### 2.3.2. HADS (M)

HADS was designed [23] to assess anxiety and depression amongst the study subjects. This scale consists of 14 items, whereby seven of the items address anxiety symptoms, and the other seven items relate to depressive symptoms. Each item has a score of 0 to 3. Hence, the scores for anxiety and depression can vary from 0 to 21, respectively. The conventional cut-off score of the scale was 11. A lower cut-off score of 8 was applied in this study to not miss out a significant fraction of the Malaysian population with anxiety and depression [24], with a sensitivity and specificity of 93.2% and 90.8%, respectively. The Malay version of this scale had a Cronbach’s alpha of 0.87 [25].

### 2.4. Statistical Analyses

Statistical Package for Social Science version 25.0 (IBM Corp., Armonk, NY, USA) was utilized to analyze the data in this study. Descriptive statistics were used to review the properties of the study population. Categorical variables were presented via frequency and percentage. Continuous variables were presented via means and standard deviation. Religious coping was then associated with anxiety and depression via Spearman rank correlation. Univariate analysis was utilized to examine the covariates associated with anxiety and depression. Covariates with *p* values of less than 0.25 from crude analysis were then tested further via multivariate analysis [26]. Depression scores (Dp) were transformed due to skewed distribution, using the formula of Ln(Dp+1)=x, for the purpose of univariate and multivariate regression analyses.

## 3. Results

### 3.1. Socio-Demographic Data

Table 1 demonstrates the socio-demographic information of the study subjects. In total, 200 HCWs participated in the study. The majority of the HCWs were between 31 and 40 years old (70.5%), followed by an age range of 20 to 30 years old (25.5%), then 41 to 50 years old (2.5%) and finally more than 51 years old (0.5%). More females (60.5%) than males (39.5%) joined the study and most of the HCWs were married (61%) and resided in urban area (87.5%). The majority of the participants of this study made up of Malay population (55.5%), followed by Chinese (31%), Indian (9.5%) and other races (4%). Most of the participants came from a middle income background (63.0%), followed by low income (23.5%) and then high income (13.5%).

HCWs were further stratified by their occupations; the majority of them were medical doctors (69.5%), followed by allied healthcare (29.5%) and nonclinicians (1%). Allied healthcare consisted of 50 registered nurses (25%), seven Assistant Medical Officers (AMOs) (3.5%), and two clinical attendants (1%). Most of the participants worked in the Emergency Department (37.5%), followed by Primary Care (Hospital setting) (20.5%) and equal percentages of HCWs from Anesthetic Department (19%) and other departments (19%) rotating to the frontline. The Primary Care Department in UMMC is an outpatient service in the hospital setting, which allows walk-in cases, chronic follow-up cases or referrals from other outpatient departments, government health clinics and private clinics. The Internal Medicine Department is made up of the minority at a percentage of 4%. A total of 46% of the study population worked in shifts. Additionally, 2% of the subjects contracted COVID-19 while 10% of the subjects were suspected of contracting COVID-19.

### 3.2. Anxiety and Depressive Symptoms amongst HCWs

Table 2 demonstrates the percentages of anxiety and depression amongst the study population. In total, 36.5% of the HCWs exhibited anxiety symptoms while 29.5% of them reported depressive symptoms; 23.5% of the HCWs had mixed anxiety and depressive symptoms. Interestingly, amidst the pandemic setting, the mean anxiety (6.64) and depressive (5.02) scores were lower than the cut-off scores of 8. Table 3 illustrates the anxiety and depressive symptoms stratified by their severity. The majority of the HCWs exhibited mild anxiety and depression (17% and 20%, respectively). Only a minority of the HCWs had severe symptoms of anxiety (3.5%) and depressive symptoms (1.5%). Table 4 revealed that the mean depression scores of this study were significantly lower than studies in both China (*p* < 0.01) and Brazil (*p* = 0.042), respectively.

### 3.3. Anxiety, Depressive Symptoms and Religious Coping Stratified by Occupations

Table 5 demonstrates the percentages of anxiety and depression amongst the study subjects, stratified by occupation, specifically medical doctors versus nurses as the nature of their job differs vastly. In total, 36.0% of the doctors and 38.0% of nurses exhibited anxiety symptoms while 33.1% of the doctors and 18.0% of the nurses reported depressive symptoms. Interestingly, the mean anxiety (6.77) and depressive (5.20) scores of the doctors were higher than the mean anxiety (6.50) and depressive scores (4.40) of the nurses. Doctors had higher percentages of moderate to severe anxiety (21.6%) and depression (13.7%) than nurses did (13.5% and 0%, respectively). However, both doctors and nurses did not show significant differences in terms of anxiety and depressive symptoms (*p* = 0.873 and *p* = 0.695).

Table 6 shows the mean scores of religious coping amongst the HCWs, stratified by their occupations—specifically doctors versus nurses. Nurses were reported to have higher mean scores of positive (25.14) and negative religious (10.78) coping than the doctors did (19.67 and 10.32, respectively). There were significant differences between the doctors and nurses in terms of positive religious coping (*p* < 0.001) and negative religious coping (*p* = 0.015).

### 3.4. Correlation between Religious Coping with Anxiety and Depression

Table 7 shows the mean scores of positive and negative religious coping, respectively, at 21.33 and 10.52, indicating that the participants adopted more positive religious coping than negative religious coping. Spearman correlation demonstrated a significant correlation between positive coping with both anxiety (*p* < 0.01) and depression (*p* < 0.05). Similarly, negative coping was found to be significantly correlated with anxiety (*p* < 0.01) and depression (*p* < 0.01). However, the effect sizes for these correlations were small.

### 3.5. Univariate and Multivariate Analyses for Covariates of Anxiety

Univariate analysis was conducted to evaluate the covariates of anxiety, utilizing a General Linear Model (GLM) (Table 8). The analysis showed that age group, department, positive religious coping and negative religious coping were crudely associated (*p* < 0.25) with anxiety. After adjusting for covariates, both positive and negative religious coping continued to be significantly associated (*p* = 0.001 and *p* < 0.001) with anxiety. With every unit of positive coping score increment, anxiety scores were predicted to reduce by 0.15 after adjusted for age group, department and negative religious coping. On the other hand, every unit of negative coping score increment could increase anxiety scores by 0.289 by adjusting for age group, department and positive religious coping. However, the effective size for these association were small. There was no significant association between remaining socio-demographic variables with anxiety. Overall, the GLM reaffirmed that positive coping was inversely associated with anxiety, while negative coping had a direct association with anxiety.

### 3.6. Univariate and Multivariate Analyses for Covariates of Depression

Univariate analysis was conducted to examine the covariates of depressive symptoms (Table 9). It showed that gender, marital status, department, shift work, positive religious coping and negative religious coping were crudely associated (*p* < 0.25) with depression. After adjusting for covariates, both positive and negative coping remained significantly associated (*p* = 0.025 and *p* < 0.001) with depression. Every unit of positive coping score increment could predict depression score reduction by 0.019 when taking into consideration variables such as gender, marital status, shiftwork, department and negative religious coping, while every unit of negative coping score increments predicted the depression score to be increased by 0.052 after adjusting for gender, marital status, shiftwork, department and positive religious coping. The effect sizes for these associations were small. Other covariates were not associated with depression. Overall, the GLM reaffirmed that positive and negative coping were significantly associated with depression, in inverse and direct relationships, respectively.

## 4. Discussion

Even in the nonpandemic setting, HCWs are at risk of facing numerous occupational hazards: enormous workload, prolonged working hours, painstaking psychological requirement and potential workplace aggression [30]. These workplace hazards increase stress as well as burnout, and potentially cause a range of mental health issues [31]. Despite these hazards, HCWs face difficulty in accessing optimal care and treatment due to the presence of stigma [32]. Amid the pandemic setting, a recent meta-analysis [5] looking at the HCWs in China, revealed a prevalence of anxiety at 23.2% and depression at 22.8%. Global data reported a prevalence ranging from 8.7% to 44.6% and 5.3% to 50.6% [2,3,4,5,6], respectively, for anxiety and depression. The prevalence results from our study fell within the prevalence range reported from previous studies.

Interestingly, the mean scores of anxiety and depression were lower than the minimum cut-off point. Our results are similar with a multicenter study conducted in India and Singapore [2], and the mean scores reported for anxiety and depression were both lower than the minimum cut-off point. Furthermore, the mean depression scores for the current study were found to be significantly lower than another international study in Brazil [29] withstanding the arduous pandemic. The predominant use of the unidirectional and paternalistic nature of the medicine in the Asian region [33] could possibly have impeded the HCWs from reporting their true levels of distress. Having an illness, particularly a mental illness, could signify vulnerability and weakness, hence prohibiting the process of seeking help or reporting the level of distress accurately. However, the same explanation does not seem to fully explicate the significant lower depression scores in this study, when compared to China, which is also in the Asian region.

Another reason for the lower scores of the mental health outcome was that the study population might have adopted higher levels of positive coping compared to negative coping methods, thus potentially giving rise to lower mean scores of anxiety and depression. Furthermore, our results revealed that the mean anxiety and depressive scores of the nurses were lower than those of the doctors, although this was not statistically significant. This finding is intriguing as previous studies [4,5] found that nursing staff were particularly at risk of developing depression and anxiety. Our results demonstrated no significant differences between the doctors and nurses for anxiety and depression, possibly owing to the nurses having significantly higher scores of positive religious coping than the doctors. However, the nurses also had significantly higher score of negative religious coping than the doctors but with its mean score differed by only 0.46. The narrow difference of mean score for negative religious coping could possibly be overwhelmed by the vast score difference in positive religious coping (5.47), hence contributing to lower mean scores of anxiety and depression in the nurses. These findings further emphasize the importance of positive religious coping in potentially predicting a better mental health outcome.

Existing studies report a stronger association between negative religious coping and psychological distress. Local studies among psychiatric patients [16] and medical students [17] showed significant correlations between negative coping with anxiety and depression but not with positive coping. Similar findings of a significant correlation between negative coping and psychological distress were also identified in a study among African Americans and Iranians [18,19]. This study, on the other hand, supported findings of significant associations between both positive and negative religious coping with anxiety and depression after adjusting for correlates by using multivariate analysis. The higher levels of positive religious coping than negative religious coping in our subjects could have contributed to the overall lower mean scores of anxiety and depression than cut-off points. Furthermore, the nurses in our study practiced higher levels of positive religious coping than the doctors, which potentially led to lower anxiety and depressive scores. The findings in our study further stress the importance of positive religious coping.

A recent study in China suggested the need to adopt positive coping methods to reduce anxiety and depression amongst HCWs amid the pandemic [6]. Additionally, another multicentric study in Italy indicated the necessity of adopting positive coping skills such as healthy lifestyle modification, stress management, problem-solving skills and communications strategies [34]. However, specific evidence of positive coping styles is not available yet. Our study revealed a significant correlation and prediction between positive religious coping and improved mental health outcomes, hence offering more plausible choices of psychological interventions for the HCWs to get through the pandemic.

Positive religious coping remains a significant coping mechanism to boost mental health, commonly via prayers, attending religious services, reading scriptures or meditation [35]. Besides enhancing mental health by reducing anxiety and depression, evidence has showed that religious coping could help in stress management [36] and traumatic experiences [37], such as in the face of this arduous pandemic. In addition, a study in China revealed that 77.8% of the hospital staff members who were infected with COVID-19 experienced psychological distress [38], hence warranting the need of psychological interventions. Therefore, HCWs diagnosed with COVID-19 will also benefit from religious coping, which enhances coping with medical illnesses, modifies psychological reactions to illness, cultivates social support and aids in making meaning in life [39]. Furthermore, the benefits of religious coping might not be limited to psychological well-being. Religion could be related to positive psychology [40], which in turn boosts the concept of global coherence and psychophysiological aspects such as the cardiovascular, respiratory, nervous and immune systems [41], which are all crucial in the maintenance of physical health amid the pandemic. However, the direct association between religious coping and psychophysiology advantages still warrants further research.

The significant correlation between religious coping and mental health outcome in this study sheds new light on ways to provide psychological interventions for the HCWs, particularly during the current pandemic. For example, the directions of the psychological intervention could be focused on enhancing positive religious coping while minimizing negative religious coping, utilizing counselling centered on religion and spirituality [42] or religious cognitive-emotional therapy [43]. Additionally, mental health professionals could integrate religious or spiritual elements into remote psychological first aid [44,45], which represents a form of emergent psychological support by the means of telecommunication to reduce the risk of cross-infection. The risk stratification of HCWs could be improved by identifying susceptible individuals with low levels of positive religious coping and high levels of negative religious coping possibly by screening via the BRCOPE. In addition, by utilizing the findings from our studies, primary prevention can be enhanced by centering in promoting positive religious coping towards predicting lower rates of anxiety and depression, such as in local or international religious campaigns. However, in the challenging times of COVID-19 pandemic, such campaigns might warrant a transformation to teleconference to reduce the risk of cross-infection.

Our study has a few limitations. Firstly, the cross-sectional design of our study renders the study of causality implausible. The sampling method was nonprobability convenient sampling, which potentially rendered the prevalence of the study population less accurate and representational of the true population of HCWs. Furthermore, the study was limited to a single university hospital, hence rendering the results from this study less generalizable. Since anxiety and depression were highly associated, future studies could aim at studying the possible mediating factors among depression, anxiety and religious coping. In addition, stress or burnout could represent a possible confounding factor to anxiety and depression, which was not included in the current research.

Furthermore, stress and burnout appeared to be crucial causes in contributing to adverse psychological outcomes in the HCWs. A recent study revealed that secondary traumatic stress and burnout could predict heightened anxiety and depressive scores amid the COVID-19 pandemic [46]. Hence, undetected stress and burnout could potentially lead to more sinister psychiatric morbidities such as depression and anxiety disorders. Including the measurement of stress or burnout among HCWs could possibly introduce new facets to the relationship amongst the variables examined. Another limitation of our study was the inability to compare the study subjects to a control group without religious beliefs. Having a control group for comparison could potentially stress the importance of having a religious belief and therefore religious coping.

## 5. Conclusions

To the knowledge of the authors, this is the first research evaluating the correlation between religious coping, anxiety and depression, specifically amongst the HCWs amidst the COVID-19 pandemic. Our data revealed that the prevalence of anxiety and depression among HCWs is comparable with previous international studies. However, the overall anxiety and depression mean scores were lower than the cut-off points, possibly related to the predominant use of positive religious coping in the HCWs. Positive and negative religious coping were both found to be significantly correlated with anxiety and depression. Enhancing positive religious coping and improving negative religious coping, by religious counseling, cognitive therapy or campaigns could aid in optimizing the mental health outcome in HCWs, especially in this strenuous and challenging time.

## Figures and Tables

**Table 1:** Socio-demographic information of healthcare workers (HCWs) (*n* = 200).

Socio-Demographic	*n* (%)
**Age range**
20–30	51 (25.5)
31–40	141 (70.5)
41–50	7 (3.5)
>51	1 (0.5)
**Gender**
Male	79 (39.5)
Female	121 (60.5)
**Race**	
Malay	111 (55.5)
Chinese	62 (31.0)
Indian	19 (9.5)
Others	8 (4.0)
**Marital status**	
Single	73 (36.5)
Married	122 (61.0)
Divorced	5 (2.5)
**Area of residence**	
Urban	175 (87.5)
Suburban	23 (11.5)
Rural	2 (1.0)
**Monthly household income (RM)**	
Low (<4000)	47 (23.5)
Middle (4001–8000)	126 (63.0)
High (>8000)	27 (13.5)
**Occupation**	
Medical Doctor	139 (69.5)
Allied Healthcare ^†^	59 (29.5)
Nonclinicians	2 (1.0)
**Department of work**	
Emergency department	75 (37.5)
Primary care (Hospital setting)	41(20.5)
Anesthesia	38 (19.0)
Internal Medicine	8 (4.0)
Other departments rotating to frontline ᵚ	38 (19.0)
**Shift work**	
Yes	92 (46.0)
No	108 (54.0)
**COVID positive status**	
Yes	4 (2.0)
No	196 (98.0)
**PUI status ᵜ**	
Yes	20 (10.0)
No	180 (90.0)

^†^ Allied healthcare: registered nurse, assistant medical officer, clinical attendant; ᵚ other departments rotating to frontline: ophthalmology, psychiatry, surgery, pediatrics, oncology, occupational medicine, rehabilitation medicine, sports medicine, otorhinolaryngology, administrative nursing; ᵜ PUI status: patient under investigation (suspected cases).

**Table 2:** Scores and cut-offs of the Hospital Anxiety and Depressive Scale (HADS) (*n* = 200).

Measures	*n* (%)	M (SD)	Prevalence Cut-Off, *n* (%)
Anxiety	73 (36.5)	6.64 (4.07)	Negative (<8) = 127 (63.5)
			Mild (8–10) = 34 (17.0)
			Moderate (11–14) = 32 (16.0)
			Severe (15–21) = 7 (3.5)
Depression	59 (29.5)	5.02 (3.93)	Negative (<8) = 141 (70.5)
			Mild (8–10) = 40 (20.0)
			Moderate (11–14) = 16 (8.0)
			Severe (15–21) = 3 (1.5)
Anxiety and depression	47 (23.5)		
Negative	115 (57.5)		

M: mean. SD: standard deviation.

**Table 3:** Severity of anxiety and depression.

Severity	Anxiety, *n* (%)	Depression, *n* (%)
Negative (<8)	127 (63.8)	141 (70.5)
Mild (8–10)	34 (17.0)	40 (20.0)
Moderate (11–14)	32 (16.0)	16 (8.0)
Severe (15–21)	7 (3.5)	3 (1.5)

**Table 4:** Comparison of anxiety and depression amongst Malaysian HCWs with identical studies from other nations.

Previous Studies and Country of Origin	Instruments Used	Sample Size Estimated Mean (E.M)	Current Study’s Sample Size with Estimated Mean (E.M)	Mean Difference (95% CI)	One Sample *t*-Test, *p*-Value
Atif K et al. [27]—Pakistan		*n* = 220 doctors	*n* = 139 doctors		
	HADS-A	7.04	6.78	−0.26 (−0.96, 0.44)	0.465
	HADS-D	4.94	5.2	0.26 (−0.47, 0.97)	0.48
Xiao Y et al. [28]—China		*n* = 205 ED doctors	*n* = 34 ED doctors		
	HADS-A	7.8	7.68	−0.12 (−01.42, 1.18)	0.853
	HADS-D	7.9	5.56	−2.34 (−3.78, −0.9)	<0.01
Schmidt DR et al. [29]—Brazil		*n* = 211 nurses	*n* = 50 nurses		
	HADS-A	6.3	6.5	0.2 (−0.89, 1.29)	0.715
	HADS-D	5.2	4.4	−0.8 (−1.57, −0.03)	0.042

HADS-A: Hospital Anxiety and Depression scale (Anxiety). HADS-D: Hospital Anxiety and Depression Scale (Depression). ED: Emergency Department. CI: confidence interval.

**Table 5:** Scores and cut-offs of anxiety and depressive symptoms, doctors versus nurses (*n* = 189).

Measures	*n* (%)	M (SD)	Prevalence Cut-off = *n* (%)	*p*-Value
**Anxiety Symptoms**				
Doctors	139 (69.5)	6.77 (4.18)	Negative (<8) = 89 (64.0)	0.873
			Mild (8–10) = 20 (14.4)	
			Moderate (11–14) = 25 (18.0)	
			Severe (15–21) = 5 (3.6)	
Nurses	50 (25.0)	6.50 (3.86)	Negative (<8) = 31 (62.0)	
			Mild (8–10) = 11 (22.0)	
			Moderate (11–14) = 6 (12.0)	
			Severe (15–21) = 3 (1.5)	
**Depressive Symptoms**				
Doctors	139 (69.5)	5.20 (4.33)	Negative (<8) = 93 (66.9)	0.695
			Mild (8–10) = 27 (19.4)	
			Moderate (11–14) = 16 (11.5)	
			Severe (15–21) = 3 (2.2)	
Nurses	50 (25.0)	4.40 (2.71)	Negative (<8) = 41 (82.0)	
			Mild (8–10) = 9 (18.0)	
			Moderate (11–14) = 0	
			Severe (15–21) = 0	

M: Mean. SD: standard deviation; *n* = 189 as 11 subjects were excluded to allow direct comparison between medical doctor and registered nurse groups.

**Table 6:** Association between occupations with positive and negative brief religious coping scales. (*n* = 189).

Measures	*n* (%)	Mean (SD)	*p-*Value
**PCOPE**			
Doctors	139 (69.5)	19.67 (7.0)	<0.001
Nurses	50 (25.0)	25.14 (2.7)	
**NCOPE**			
Doctors	139 (69.5)	10.32 (4.28)	0.015
Nurses	50 (25.0)	10.78 (3.0)	

SD: standard deviation. *n* = 189 as 11 subjects were excluded to allow direct comparison. between medical doctor and registered nurse groups. PCOPEs: Positive Brief Religious Coping Scales. NCOPEs: Negative Brief Religious Coping Scales.

**Table 7:** Spearman correlation between Brief Religious Coping Scale, anxiety and depression.

	Mean (SD)	PCOPE	NCOPE	HADS M	HADS M
				(Anxiety)	(Depression)
PCOPE	21.33 (6.53)	1			
NCOPE	10.52 (3.93)	0.194 **	1		
HADS M (anxiety)	6.64 (4.07)	−0.224 **	0.225 **	1	
HADS M (depression)	5.02 (3.93)	−0.164 *	0.242 **	0.727 **	1

SD: standard deviation. * *p* < 0.05. ** *p* < 0.01. PCOPE: positive religious coping. NCOPE: negative religious coping. HADS M: Hospital Anxiety and Depressive Scale Malay translation.

**Table 8:** General linear model (GLM) on covariates associated with anxiety (*n* = 199).

Variables	Univariate Analysis			Multivariate Analysis		
	b^1^ (95% CI)	*p* Value	Partial eta^2^	b^2^ (95% CI)	*p* Value	Partial eta^2^
**Age group**		**0.245**	**0.007**		**0.093**	**0.015**
Just entered work ᵚ	−0.767 (−2.064, 0.530)	0.245	0.007	−1.080 (−2.344, 0.183)	0.093	0.015
Prime working age						
**Gender**		**0.517**	**0.002**			
Male	0.382 (−0.778, 1.542)	0.517	0.002			
Female						
**Race**		**0.298**	**0.019**			
Malay	0.663 (−2.258, 3.585)	0.655	0.001			
Chinese	1.801 (−1.200, 4.802)	0.238	0.007			
Indian	1.270 (−20.94, 4.633)	0.457	0.003			
Others						
**Marital status**		**0.455**	**0.008**			
Single	1.934 (−1.766, 5.634)	0.304	0.005			
Married	2.235 (−1.418, 5.887)	0.229	0.007			
Divorced						
**Area of residence**		**0.386**	**0.010**			
Urban	2.069 (−3.618, 7.756)	0.474	0.003			
Suburban	3.109 (−2.787, 9.004)	0.300	0.005			
Rural						
**Monthly income**		**0.435**	**0.008**			
Low	−0.923 (−2.879, 1.033)	0.353	0.004			
Middle	−0.058 (−1.782, 1.666)	0.947	0.000			
High						
**Occupation**		**0.410**	**0.003**			
Doctor	0.515 (−0.716, 1.745)	0.410	0.003			
Nondoctor ᵝ						
**Department**		**0.054**	**0.038**		**0.058**	**0.038**
ED ᶱ	0.496 (−1.077, 2.069)	0.535	0.002	−0.072 (−1.170, 1.026)	0.634	0.001
Primary care	−0.611 (−2.400, 1.179)	0.502	0.002	−0.437 (−1.724, 0.851)	0.140	0.011
Ward care ᶶ	1.703 (−0.029, 3.434)	0.054	0.019	0.381 (−0.851, 1.614)	0.301	0.006
Other departments						
**Shift work**		**0.812**	**0.000**			
No	−0.138 (−1.278, 1.002)	0.812	0.000			
Yes						
**PUI status ᵞ**		**0.617**	**0.001**			
No	−0.480 (−2.368, 1.409)	0.617	0.001			
Yes						
**PCOPE ᵟ**	−0.120 (−0.206, −0.035)	**0.006**	**0.038**	−0.150 (−0.234, −0.066)	**0.001**	**0.061**
**NCOPE ᵠ**	0.240 (0.099, 0.380)	**0.001**	**0.054**	0.289 (0.151, 0.428)	**0.000**	**0.081**

ᵚ Just entered work: age 20–30. Prime working age: age 31–50. ᵝ Nondoctor: allied healthcare + nonclinician. ᶱ ED: Emergency Department. ᶶ Ward care: anesthetic + internal medicine. ᵞ PUI: patient under investigation/suspected cases. ᵟ PCOPE: positive religious coping. ᵠ NCOPE: negative religious coping. *n* = 199: Excluded one case of approaching retirement age of 51–64. Variables with *p* values < 0.25 were preserved for multivariate analyses. b^1^ indicates crude regression coefficient; b^2^ represents regression coefficient (adjusted). CI indicates confidence interval. Partial eta^2^: effect size (estimated). Bolded *p* values and partial eta^2^ represent comparison between different variable groups while nonbolded values represent comparison values within the same variable.

**Table 9:** GLM on covariates associated with transformed depression (*n* = 199).

Variables	Univariate Analysis			Multivariate Analysis		
	b^1^ (95% CI)	*p*-Value	Partial eta^2^	b^2^ (95% CI)	*p*-Value	Partial eta^2^
**Age group**		**0.639**	**0.001**			
Just entered work ᵚ	−0.058 (−0.301, 0.185)	0.639	0.001			
Prime working age						
**Gender**		**0.157**	**0.010**		**0.703**	**0.001**
Male	0.155 (−0.060, 0.371)	**0.157**	**0.010**	0.042 (−0.174, 0.258)	0.703	0.001
Female						
**Race**		**0.532**	**0.011**			
Malay	0.338 (−0.209, 0.886)	0.224	0.008			
Chinese	0.413 (−0.149, 0.976)	0.149	0.011			
Indian	0.392 (−0.238, 1.023)	0.221	0.008			
Others						
**Marital status**		**0.034**	**0.034**		**0.141**	**0.021**
Single	0.804 (0.122, 1.485)	0.021	0.027	0.552 (−0.119, 1.223)	0.106	0.014
Married	0.885 (0.212, 1.558)	0.010	0.033	0.639 (−0.023, 1.301)	0.058	0.019
Divorced						
**Area of residence**		**0.781**	**0.003**			
Urban	−0.212 (−1.278, 0.854)	0.695	0.001			
Suburban	−0.112 (−1.217, 0.994)	0.842	0.000			
Rural						
**Monthly income**		**0.898**	**0.001**			
Low	0.032 (−0.334, 0.399)	0.863	0.000			
Middle	−0.028 (−0.351, 0.296)	0.867	0.000			
High						
**Occupation**		**0.802**	**0.000**			
Doctor	−0.029 (−0.259, 0.201)	0.802	0.000			
Nondoctor ᵝ						
**Department**		**0.065**	**0.036**		**0.192**	**0.025**
ED ᶱ	0.147 (−0.147, 0.441)	0.326	0.005	0.105 (−0.195, 0.405)	0.491	0.003
Primary care	−0.142 (−0.476, 0.193)	0.405	0.004	−0.216 (−0.563, 0.130)	0.220	0.008
Ward care ᶶ	0.265 (−0.058, 0.589)	0.108	0.013	0.138 (−0.177, 0.453)	0.390	0.004
Other departments						
**Shift work**		**0.225**	**0.007**		**0.756**	**0.001**
No	−0.131 (−0.343, 0.081)	0.225	0.007	0.041 (−0.221, 0.304)	0.756	0.001
Yes						
**PUI status ᵞ**		**0.983**	**0.000**			
No	−0.004 (−0.357, 0.349)	0.983	0.000			
Yes						
**PCOPE ᵟ**	−0.015 (−0.031, 0.002)	**0.076**	**0.016**	−0.019(−0.036, 0.002)	**0.025**	**0.026**
**NCOPE ᵠ**	0.047 (0.021, 0.073)	**0.000**	**0.060**	0.052 (0.025, 0.078)	**0.000**	**0.072**

ᵚ Just entered work: age 20–30. Prime working age: age 31–50. ᵝ Nondoctor: allied healthcare + nonclinician. ᶱ ED: Emergency Department. ᶶ Ward care: anesthetic + internal medicine. ᵞ PUI: patient under investigation/suspected cases. ᵟ PCOPE: positive religious coping. ᵠ NCOPE: negative religious coping. Transformed depression: transformed scores of depression using the formula Ln (Dp + 1), as depression scores were skewed. *n* = 199: excluded one participant who was approaching retirement age (51–64). Variables with *p* values < 0.25 were preserved for multivariate analyses. b^1^ indicates a crude regression coefficient; b^2^ indicates a regression coefficient (adjusted). CI represents confidence intervals. Partial eta^2^: effect size (estimated). Bolded *p* values and partial eta^2^ represent comparison between different variable groups, while nonbolded values indicate comparison values within the same variable.

## Data Availability

The data presented in this study are available on request from the corresponding author. The data are not publicly available due to privacy and ethical issues.
